# Plastid Genome-Based Phylogeny Pinpointed the Origin of the Green-Colored Plastid in the Dinoflagellate *Lepidodinium chlorophorum*

**DOI:** 10.1093/gbe/evv060

**Published:** 2015-04-02

**Authors:** Ryoma Kamikawa, Goro Tanifuji, Masanobu Kawachi, Hideaki Miyashita, Tetsuo Hashimoto, Yuji Inagaki

**Affiliations:** ^1^Graduate School of Global Environmental Studies and Graduate School of Human and Environmental Studies, Kyoto University, Japan; ^2^Graduate School of Life and Environmental Sciences, University of Tsukuba, Ibaraki, Japan; ^3^The National Institute for Environmental Studies, Tsukuba, Ibaraki, Japan; ^4^Center for Computational Sciences, University of Tsukuba, Ibaraki, Japan

**Keywords:** genome reduction, *Lepidodinium*, Pedinophyceae, secondary plastids, plastid replacement

## Abstract

Unlike many other photosynthetic dinoflagellates, whose plastids contain a characteristic carotenoid peridinin, members of the genus *Lepidodinium* are the only known dinoflagellate species possessing green alga-derived plastids. However, the precise origin of *Lepidodinium* plastids has hitherto remained uncertain. In this study, we completely sequenced the plastid genome of *Lepidodinium chlorophorum* NIES-1868*.* Our phylogenetic analyses of 52 plastid-encoded proteins unite *L. chlorophorum* exclusively with a pedinophyte, *Pedinomonas minor*, indicating that the green-colored plastids in *Lepidodinium* spp. were derived from an endosymbiotic pedinophyte or a green alga closely related to pedinophytes. Our genome comparison incorporating the origin of the *Lepidodinium* plastids strongly suggests that the endosymbiont plastid genome acquired by the ancestral *Lepidodinium* species has lost genes encoding proteins involved in metabolism and biosynthesis, protein/metabolite transport, and plastid division during the endosymbiosis. We further discuss the commonalities and idiosyncrasies in genome evolution between the *L. chlorophorum* plastid and other plastids acquired through endosymbiosis of eukaryotic photoautotrophs.

## Introduction

Plastids are photosynthetic organelles that are found in diverse lineages, scattered across the vast diversity of eukaryotes (Keeling 2004; Kim and Archibald 2009). A cyanobacterium-eukaryote endosymbiosis, called primary endosymbiosis, produced the first photosynthetic eukaryote (De Clerck et al. 2012), which subsequently diverged into three lineages with “primary plastids,” namely red algae, green algae/land plants, and glaucophytes (Rodriguez-Ezpeleta et al. 2005; Keeling 2010). Plastids further spread into diverse eukaryotes through multiple events involving the uptake and retention of either a green or red alga by a heterotrophic eukaryote, a phenomenon referred to as secondary endosymbiosis (Douglas 1998; Archibald 2012). Henceforth, we refer to the plastids that emerged from secondary endosymbioses as “secondary plastids.” Euglenophytes and chlorarachniophytes acquired their plastids from different endosymbiotic green algae, a prasinophyte and a member of core chlorophytes, respectively (Rogers et al. 2007; Turmel, Gagnon, et al. 2009). Thus, green alga-involved secondary endosymbiosis has occurred multiple times (at least twice) in eukaryotes. On the other hand, the vast majority of photosynthetic dinoflagellates, photosynthetic lineages basal to apicomplexan parasites (i.e., *Chromera velia* and *Vitrella brassicaformis*), haptophytes, cryptophytes, and heterokont algae, possesses red alga-derived plastids; however, it remains controversial whether their red alga-derived plastids result from a single or multiple endosymbiotic events (Archibald 2009; Keeling 2009).

Dinoflagellates are a part of a large microeukaryotic assemblage termed Alveolata, and about 50% of the known species are photosynthetic (Taylor 1987). The vast majority of the photosynthetic species possesses red alga-derived secondary plastids, which contain chlorophylls (Chls) *a* and *c*, plus a dinoflagellate-specific carotenoid, peridinin (Schnepf and Elbrachter 1999). However, some dinoflagellate species replaced the original (peridinin-containing) plastids with others bearing distinct carotenoid compositions. For instance, plastids in members of the genera *Karenia*, *Karlodinium*, and *Takayama* contain Chls *a* and *c*, plus 19′ hexanoyloxyfucoxanthin, instead of peridinin. The 19′ hexanoyloxyfucoxanthin-containing plastids most likely originated from an endosymbiotic haptophyte (Tengs et al. 2000; Keeling 2004). Likewise, *Durinskia baltica* and other closely related species possess diatom-derived plastids containing Chls *a* and *c*, plus fucoxanthin (Jeffrey et al. 1975; Keeling 2004).

The dinoflagellate genus *Lepidodinium* possesses plastids containing Chls *a* and *b*, which are believed to be remnants of an endosymbiotic green alga (Watanabe et al. 1990; Matsumoto et al. 2012). Prior to this study, there were two lines of studies on *Lepidodinium chlorophorum*: 1) A survey of green algal genes endosymbiotically transferred to the host genome, and 2) an investigation of the precise origin of the *Lepidodinium* plastids. First, surveys of the host nuclear transcripts in *L. chlorophorum* detected putative plastid genes of both green algal and peridinin-containing dinoflagellate origins. In organismal phylogenies, *L. chlorophorum* affiliates with lineages possessing peridinin-containing plastids, suggesting that the original plastid was replaced by a green algal endosymbiont on the branch leading to *Lepidodinium* spp. (e.g., Saldarriaga et al. 2001; Shalchian-Tabrizi et al. 2006). Thus, Takishita et al. (2008) and Minge et al. (2010) proposed that 1) the putative “green algal” genes were transferred from the green algal endosymbiont nucleus, and 2) the “typical dinoflagellate” plastid genes were present in the host nucleus prior to the acquisition of the green algal endosymbiont. On the other hand, studies analyzing plastid genes and pigment composition failed to resolve the precise origin of the *Lepidodinium* plastids (Takishita et al. 2008; Matsumoto, Shinozaki, et al. 2011; Matsumoto, Ishikawa, et al. 2011; Matsumoto et al. 2012). So far, a phylogenetic study of 11 plastid genes by Matsumoto, Shinozaki, et al. (2011) narrowed down the origin of the *Lepidodinium* plastids to core chlorophytes, comprising Ulvophyceae, Trebouxiophyceae, Chlorophyceae, Pedinophyceae, and Chlorodendrophyceae (Leliaert et al. 2012).

We report here the complete plastid genome of *L. chlorophorum*, along with phylogenetic analyses based on plastid genome data. Our phylogenetic analyses of 52 genes conserved across green algal plastid genomes suggests a robust affinity between *L. chlorophorum* and a pedinophyte, *Pedinomonas minor*. Thus, we conclude that the plastids in *Lepidodinium* spp. were derived from a pedinophyte (or an endosymbiotic alga closely related to pedinophytes). Our determination of the precise origin of *Lepidodinium* plastids enables us to discuss the possible evolution of the plastid genome in the endosymbiont. We further discuss the commonalities and idiosyncrasies in genome evolution between the *L. chlorophorum* plastid and other “noncanonical” dinoflagellate plastids lacking peridinin.

## Materials and Methods

### Plastid Genome Sequencing

Cells of *L. chlorophorum* NIES-1868 were cultivated in MNK medium (Matsumoto, Shinozaki, et al. 2011). Total DNA, extracted by the CTAB method (Kamikawa et al. 2009), was sequenced on an Illumina HiSeq 2000 platform. In total, 136 million paired-end reads were obtained. The first and last five bases of each read were trimmed using the FASTX-Tool kit (Ver.0.0.13) (http://hannonlab.cshl.edu/fastx_toolkit/, last accessed April 5, 2015). Approximately 130 million paired-end reads were kept, all of which had greater than 75% of bases above a quality score of 20. Genome contigs were generated using Ray (Boisvert et al. 2012). Genome scaffolding with preassembled contigs was done using SSPACE (Boetzer et al. 2011). In total, 18 contigs for putative plastid genome sequences were identified based on sequence similarity to previously determined plastid genomes. Gapped regions were filled by polymerase chain reaction and subsequent Sanger sequencing. Annotation was performed by MFANNOT (http://megasun.bch.umontreal.ca/cgi-bin/mfannot/mfannotInterface.pl, last accessed October 30, 2014), RNAWEASEL (http://megasun.bch.umontreal.ca/RNAweasel/, last accessed September 23, 2014), tRNAscan-SE (Lowe and Eddy 1997), and BLAST (Basic Local Alignment Search Tool) searches of the GenBank database (http://www.ncbi.nlm.nih.gov/, last accessed October 30, 2014). The nucleotide sequence of the *L. chlorophorum* plastid genome was deposited to DNA Data Bank of Japan (accession no. LC008447).

### Phylogenetic Analysis

For phylogenetic analysis assessing the origin of the *L. chlorophorum* plastid, we selected 52 plastid-encoded proteins shared among the plastid genomes of 7 chlorophytes, 5 trebouxiphytes, 4 ulvophytes, a pedinophyte (*P. minor*), 6 prasinophytes, a chlorarachniophyte (*Bigelowiella natans*), a euglenophyte (*Euglena gracilis*), and *L. chlorophorum* (26 taxa in total; supplementary table S1, Supplementary Material online). We considered plastids of the prasinophytes and the euglenophyte as the outgroup. All genome data analyzed here, except those of *L. chlorophorum*, were available as of August 2014. Amino acid sequences of each protein were aligned and trimmed manually. We analyzed 52 single protein alignments individually by the maximum-likelihood (ML) method, with the LG (Le and Gascuel 2008) + Γ + F model, and detected no apparent signal of nonvertical gene inheritance in any of the single-protein alignments (data not shown). We concatenated all the single-protein alignments into a single 52-protein alignment (8,917 amino acid positions in total), and subjected it to the ML method with the LG + Γ + F model. The ML tree was heuristically searched from ten distinct maximum parsimony (MP) trees. In ML bootstrap analyses (100 replicates), a heuristic tree search was performed from a single MP tree per replicate. The ML phylogenetic analyses described above were conducted by RAxML ver. 7.2.8 (Stamatakis 2006).

The 52-protein alignment was also analyzed by PhyloBayes ver. 3.3 under the CAT-GTR + Γ model (Lartillot et al. 2009). Two parallel Markov Chain Monte Carlo chains were run for 13,000 cycles, in which 500,000 trees and log-likelihoods (lnLs) were sampled. We considered independent runs converged when the maximum discrepancy observed across all bipartitions was less than 0.3, and the effective sample size was greater than 100 (Lartillot et al. 2009). The first 2,500 cycles were discarded as burn-in, and the remaining trees were summarized to obtain Bayesian posterior probabilities (BPPs).

We prepared two extra 52-protein alignments, and subjected them to the ML method as described above. The second 52-protein alignment was generated from the original alignment by adding two streptophytes and four prasinophytes (32 taxa and 8,917 amino acid positions in total). The third 52-protein alignment was identical to the original alignment, except that *B. natans* and *E. gracilis* were excluded (24 taxa and 8,917 amino acid positions in total).

## Results

### General Features of the *L. chlorophorum* Plastid Genome

**Fig. 1.—:**
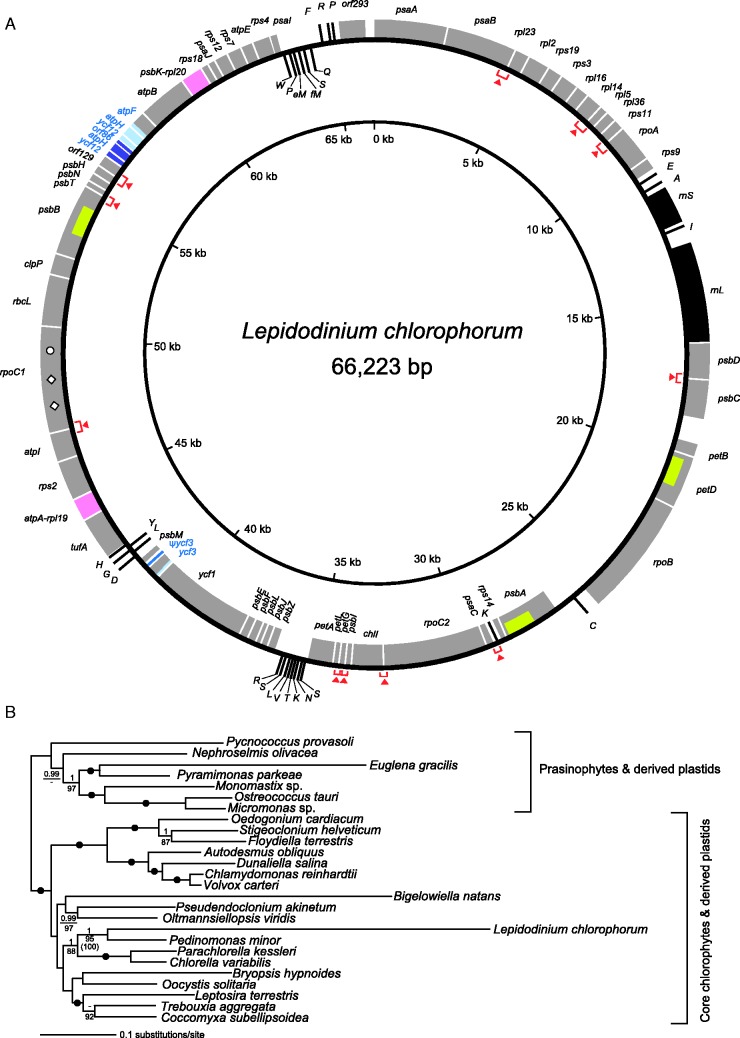
Complete plastid genome of *Lepidodinium chlorophorum* and ML phylogeny inferred from an alignment comprising 52 plastid proteins. (*A*) Physical map of the *L. chlorophorum* plastid genome. Protein-coding and rRNA-coding regions are shown by gray and closed boxes, respectively; tRNAs and pseudogenes are shown by lines. Tandemly duplicated regions were highlighted in light blue and dark blue. Two ORFs generated by gene fusion are colored in pink (see supplementary fig. S1, Supplementary Material online, for the details). Red arrowheads indicate physically overlapping coding regions (see supplementary fig. S2, Supplementary Material online, for the details). Group II introns are depicted as yellowish green boxes (see supplementary fig. S3, Supplementary Material online, for the details). A single UAA and two UAG codons found within the *rpoC1* coding region are shown by an open circle and open diamonds, respectively (see supplementary fig. S4, Supplementary Material online, for the details). (*B*) Phylogenetic analysis exploring the origin of the *L. chlorophorum* plastid. The alignment comprises 52 plastid proteins (8,917 amino acid positions in total) sampled from 26 genomes of 23 green algae and 3 green alga-derived plastids. Phylogenetic analyses were performed by both ML and Bayesian frameworks. As both methods reconstructed very similar trees, only the ML tree is shown here. BPPs and ML bootstrap values (MLBPs) are shown above and below the corresponding nodes. MLBPs less than 80% and BPPs less than 0.95 are omitted from the figure. Dots correspond to MLBPs of 100% and BPPs of 1.00. The MLBP from the analysis excluding rapidly evolving *Bigelowiella natans* and *Euglena gracilis* is shown in parentheses.

The complete sequence of the *L. chlorophorum* plastid genome can be mapped as a circular molecule of 66,223 bp (fig. 1*A*). The overall A+T content is 65.4%, and approximately 84% of the genome comprises coding region. We identified two ribosomal RNA (rRNA) genes and 27 transfer RNA (tRNA) genes, which are sufficient to translate all of the amino acid codons (supplementary table S2, Supplementary Material online). The *L. chlorophorum* plastid genome likely utilizes a deviant genetic code in which AUA is assigned as methionine as reported in Matsumoto, Ishikawa, et al. (2011). Out of 63 open reading frames (ORFs), putative functions for 60 ORFs were assigned based on sequence similarity (fig. 1*A*). Two pairs of genes appeared to be fused with each other (colored in pink in fig. 1*A*; see supplementary fig. S1, Supplementary Material online, for the details). All of the plastid genes listed above (except three ORFs of unknown function) had been found in green algal plastid genomes sequenced prior to this study. Notably, the *L. chlorophorum* plastid genome was found to possess only a single rRNA operon, contrasting to typical plastid genomes with two rRNA operons as a part of inverted repeats (IRs). We observed 11 overlapping gene-pairs (indicated by red arrowheads in fig. 1*A*; see supplementary fig. S2, Supplementary Material online, for the details). Three genes were found to possess introns (yellowish green boxes shown in fig. 1*A*), which were predicted as group II introns based on the putative secondary structure (supplementary fig. S3, Supplementary Material online; Note that no obvious ORF was found in any of the three introns, although typical group II introns carry an intronic ORF coding reverse transcriptase, maturase, and endonuclease domains [e.g., Lambowitz and Zimmerly 2004; Kamikawa et al. 2009]). We suspect that a gene-duplication has created a tandem array of *ycf3*-like regions (ψ*ycf3*; colored in light blue and dark blue in fig. 1*A*); one seemingly encodes a functional protein, and the other is most likely nonfunctional due to a large deletion (supplementary fig. S4, Supplementary Material online). Likewise, a region containing the 5′ terminal portion of *atpF*, and the entire *atpH* and *ycf12* was likely duplicated in tandem (colored in light blue and dark blue in fig. 1*A*; Note that *orf86* corresponds to the partial *atpF*). It remains uncertain whether *rpoC1* is functional, as the corresponding ORF is interrupted by one UAG codon and two UAA codons (shown by an open circle and open diamonds, respectively, in fig. 1*A*; see supplementary fig. S5, Supplementary Material online, for the details). Note that both UAG and UAA codons are used as translation termination signal for other ORFs (supplementary table S2, Supplementary Material online). We confirmed that these in-frame termination codons were present on the corresponding mRNA (data not shown), indicating that these “stop” codons were not edited posttranscriptionally. Thus, *rpoC1* is transcriptionally active but may be pseudogenic, raising the possibility that a functional gene copy exists in the host genome.

### Phylogenetic Affinity between the Plastids of *L. chlorophorum and P. minor*

The origin of the *L. chlorophorum* plastids was assessed by phylogenetic analyses of the 52-protein alignment (fig. 1*B*). The overall phylogenetic relationship among green algal (and green alga-derived) plastids recovered in this study was compatible with those presented in previously published phylogenetic studies (e.g., Smith et al. 2011; Lemieux et al. 2014). However, our 52-protein analyses successfully resolved the precise origin of the *Lepidodinium* plastid genome with high statistical support. In the ML analysis with the LG + Γ + F model, *L. chlorophorum* grouped with *P. minor *with an ML bootstrap value (MLBP) of 95% (fig. 1*B*). The phylogenetic relationship between *L. chlorophorum* and *P. minor *was reconstructed with an MLBP of 97% in the analyses of the second 52-protein alignment expanding the outgroup taxa (supplementary fig. S6, Supplementary Material online).

We were concerned whether the position of *L. chlorophorum* was severely biased in tree reconstruction due to the rapidly evolving nature of its plastid-encoded proteins. To test the above possibility, we then reassessed the affinity between *L. chlorophorum* and *P. minor* by applying a site-heterogeneous substitution model claimed to be robust against long-branch attraction artifacts (Lartillot et al. 2007; Rodriguez-Ezpeleta et al. 2007). We subjected the 52-protein alignment to Bayesian analysis with a site-heterogeneous model (CAT-GTR + Γ). As the trees inferred by ML analysis with the LG + Γ + F model and by Bayesian analysis with the CAT-GTR + Γ model were very similar to each other, only BPPs were presented in figure 1*B*. Significantly, the Bayesian tree inferred under the site-heterogeneous model recovered the clade of *L. chlorophorum* and *P. minor* with a BPP of 1.00 (fig. 1*B*). Further, the relationship between *L. chlorophorum* and *P. minor* was also investigated by excluding other long-branch sequences from the 52-protein alignment. *Lepidodinium chlorophorum* and *P. minor* remained as a clade with an MLBP of 100% in the third ML analysis*.* As the tree topology is basically consistent with figure 1*B*, only the MLBP of the node uniting *L. chlorophorum* and *P. minor* is presented in parentheses (fig. 1*B*). As both ML and Bayesian analyses of the 52-protein alignments consistently recovered the specific affinity between *L. chlorophorum* and *P. minor*, with high statistical support, we conclude that an endosymbiotic pedinophyte or green alga closely related to pedinophytes gave rise to the current plastid in *L. chlorophorum*.

## Discussion

The precise origin of the green-colored plastids in *Lepidodinium* spp. had been controversial since the initial description of these dinoflagellates (Watanabe et al. 1990). Our phylogenetic analyses, based on plastid-encoded proteins, pinpointed the progenitor of *Lepidodinium* plastids as a pedinophyte or an alga exclusively related to pedinophytes. Unfortunately, the results presented above cannot exclude the latter possibility. However, to our knowledge, there is little evidence implying any undescribed green alga/algae specifically related to the known pedinophytes; for this reason, we favor the explanation that *Lepidodinium* plastids are descended from a pedinophyte. These similar but distinguishable scenarios for the origin of *Lepidodinium* plastids should be revisited when plastid genome data from a broader collection of core chlorophytes become available.

Some populations of a dinoflagellate, *Noctiluca scintillans*, in Southeast Asian tropical seawater have been documented to possess a pedinophyte endosymbiont, *Pedinomonas noctilucae* (Sweeney 1971). *Pedinomonas noctilucae* is dispensable for *N. scintillans*, but supplies organic matter and facilitates host survival during starvation (Hansen et al. 2004; Saito et al. 2006). If *Lepidodinium* plastids are truly of pedinophyte origin, two independent dinoflagellate lineages have acquired pedinophytes as endosymbionts. Although the integration level of endosymbionts into the host system is apparently different between *Lepidodinium* spp. and *N. scintillans*, it is worth investigating whether those two independent partnerships were established on common genetic, physiological, and/or environmental grounds.

**Fig. 2.—:**
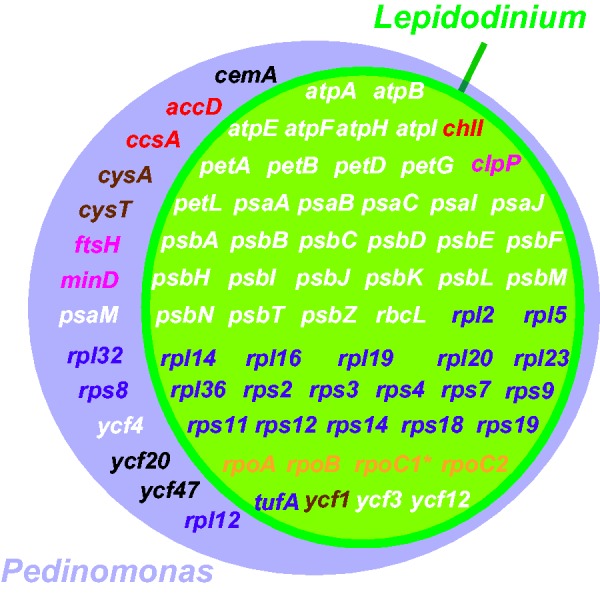
Venn diagram of the gene contents in the plastid genomes of *Lepidodinium chlorophorum *and *Pedinomonas minor. *This diagram illuminates that the protein-coding gene set in *L. chlorophorum* is the subset of that of *P. minor*. *rpoC1* is highlighted by an asterisk, as this ORF is disrupted by multiple stop codons. Genes encoding proteins involved in metabolism, plastid division, translation, transcription, transport, and photosynthesis are shown in red, magenta, purple, yellow, brown, and white, respectively.

To decipher how the plastid genome was modified after the green algal endosymbiosis, we consider the *P. minor* plastid genome as a surrogate for the plastid genome of the green algal endosymbiont captured by the ancestral *Lepidodinium* species (table 1). Here, we focus on 1) the evolution of plastid genetic code and 2) genome reduction. First, the AUA codon was reassigned from isoleucine to methionine in the plastid genome after the uptake of the endosymbiont by the ancestral *Lepidodinium* species (Matsumoto, Ishikawa, et al. 2011), as the AUA codon specifies isoleucine in the *P. minor *plastid genome, along with other plastid genomes. A distinct type of deviant genetic code, in which the UGA codon assigns tryptophan, was reported in the plastid of an alveolate lineage distantly related to dinoflagellates (i.e., the chromerid *C. velia* and apicomplexan parasites; Moore et al. 2008). We may identify additional plastid genomes using deviant genetic codes by sequencing plastid genomes in more phylogenetically diverse eukaryotes; this would also help us to understand the evolution of plastid genetic codes more precisely. Second, plastid genome comparisons revealed postendosymbiotic genome reduction in *L. chlorophorum*. Overall, the *P. minor* plastid genome (∼98 kb) is approximately 1.5 times larger than the *L. chlorophorum* plastid genome (∼66 kb), regardless of the presence of group II introns and gene duplications (table 1). Gene density appeared to be higher in the *L. chlorophorum* plastid genome than the *P. minor* plastid genome; the proportion of intergenic regions in the former is smaller than in the latter (13.3 and 25.6%, respectively; Turmel, Otis, et al. 2009; this study; table 1). Eleven physically overlapping gene-pairs, as well as two gene fusions, contribute to genome compaction in the *L. chlorophorum* plastid genome; this is considerably more than the two overlapping gene-pairs and complete absence of gene fusion in the *P. minor* plastid genome (table 1). The size difference between the *L. chlorophorum* and *P. minor* plastid genomes also coincides with a difference in gene content (fig. 2; table 1). After the endosymbiosis, the *L. chlorophorum* plastid genome discarded 14 functionally assignable ORFs, many of which are involved in plastid division, protein/metabolite transport, and metabolism (fig. 2). Similar gene losses during secondary endosymbioses were proposed for the plastids in euglenophytes (Turmel, Gagnon, et al. 2009; Hrdá et al. 2012), chlorarachniophytes (Rogers et al. 2007; Tanifuji et al. 2014), heterokont algae (Oudot-Le Secq et al. 2007; Cattolico et al. 2008), cryptophytes (Khan et al. 2007), haptophytes (Sánchez Puerta et al. 2005), and photosynthetic lineages basal to apicomplexan parasites (Janouškovec et al. 2010). Unfortunately, our current knowledge is insufficient to clarify the precise driving force(s) for the gene losses commonly observed among these plastids. Finally, the reductive trend in the *L. chlorophorum* plastid genome is seemingly congruent with the absence of IRs (fig. 1*A* and table 1). However, it is difficult to conclude that the IRs were lost as a part of endosymbiotic genome reduction, as “IR-lacking” plastid genomes that are larger than the *L. chlorophorum* plastid genome have been reported. For instance, the IR-lacking plastid genome of *Leptosira terrestris* is 195 kb in size (de Cambiaire et al. 2007). There are also examples of plastid genomes bearing IRs that are similar in size to the *L. chlorophorum* plastid genome: IRs are present in the plastid genome of *B. natans* (69 kb; Rogers et al. 2007).

**Table 1:** General Features of the Plastid Genomes in *Lepidodinium chlorophorum* (This Study) and *Pedinomonas minor* (Turmel, Otis, et al. 2009)

	*Lepidodinium chlorophorum*	*Pedinomonas minor*
Length (bp)	66,223	98,340
IRs	Absent	Present
AT content (%)	65.4	65.2
Intergenic regions (%)	13.3	25.6
Protein/tRNA/rRNA genes^a^	60^b^/27/2	74^b^/28/3
Introns	3^c^ (*psbA*, *psbB*, and *petD*)	None
Physically overlapping gene pairs	*rpl23-rpl2*, *rpl14-rpl5*, *rps11-rpoA*, *psaC-trnK*(cuu), *psbB-psbT*, *psbD-psbC*, *petL-petG*, *petG-psbI*, *chlI-rpoC2*, *atpI-rpoC1*, and *psbH-orf129*	*psbD-psbC* and *cysA-trnG*
Fused ORFs	*rpl19-atpA*, *psbK-rpl20*	None
Duplications	*ycf12-atpH-atpF* and *ycf3*	None
Genetic code	Deviant (AUA for methionine)	Standard

^a^Duplicated gene copies were counted only once.

^b^Unconserved, species-specific ORFs were excluded.

^c^All the introns found in the *L. chlorophorum* plastid genome are group II.

The data of the *P. minor* plastid genome were retrieved from Turmel, Otis, et al. (2009).

Besides the pedinophyte-derived plastids (or those derived from a green alga related to pedinophytes) in *Lepidodinium* spp., “noncanonical” plastids derived from haptophyte and diatom endosymbionts are known among dinoflagellates (see Introduction). Reflecting the separate origins of the three noncanonical plastids in dinoflagellates, their genomic features vary considerably. The genome of the haptophyte-derived plastid in *Karlodinium veneficum* lacks 39 protein-coding genes that are present in the plastid genome of the free-living haptophyte *Emiliania huxleyi*, suggesting that it underwent more extensive gene loss than *L. chlorophorum* did (Gabrielsen et al. 2011). Furthermore, mRNA editing and poly-U addition to the 3′-end of mRNA in the haptophyte-derived plastids (Dorrell and Howe 2012; Jackson et al. 2013; Richardson et al. 2014) have not been found in the green alga-derived plastid of *L. chlorophorum* or the diatom-derived dinoflagellate plastid (Matsumoto, Ishikawa, et al. 2011; Richardson et al. 2014). Unlike the noncanonical plastids in *L. chlorophorum* and *K*. *veneficum*, the genomes of diatom-derived plastids in *D. baltica* and *Kryptoperidinium foliaceum* were found to retain similar gene contents to those of free-living diatoms (Imanian et al. 2010), suggesting that no significant gene loss occurred during this endosymbiosis. As briefly overviewed above, it is difficult to extract the commonalities shared among the noncanonical dinoflagellate plastids with distinct evolutionary backgrounds. Rather, future studies need to address the reason(s) why the dinoflagellate systems can welcome diverse eukaryotic algae as endosymbionts, and integrate them as new plastids.

## Supplementary Material

Supplementary tables S1 and S2 and figures S1–S6 are available at *Genome Biology and Evolution* online (http://www.gbe.oxfordjournals.org/).

Supplementary Data
