# Supplementary material for: Plastid Genome-Based Phylogeny Pinpointed the Origin of the Green-Colored Plastid in the Dinoflagellate Lepidodinium chlorophorum
Source: Genome Biol Evol. 2015 Apr 2;7(4):1133–40. doi: 10.1093/gbe/evv060 (PMC4419806; doi:10.1093/gbe/evv060)
Supplement: Supplementary Data [file supp_7_4_1133__index.html]

Plastid Genome-Based Phylogeny Pinpointed the Origin of the Green-Colored Plastid in the Dinoflagellate Lepidodinium chlorophorum — Supplementary Data 

# Plastid Genome-Based Phylogeny Pinpointed the Origin of the Green-Colored Plastid in the Dinoflagellate *Lepidodinium chlorophorum*

## Supplementary Data

files

**Files in this Data Supplement:**

- Supplementary Data - pdf file
- Supplementary Data - pdf file
- Supplementary Data - docx file
