# Supplementary material for: Plastid Genome-Based Phylogeny Pinpointed the Origin of the Green-Colored Plastid in the Dinoflagellate Lepidodinium chlorophorum
Source: Genome Biol Evol. 2015 Apr 2;7(4):1133–40. doi: 10.1093/gbe/evv060 (PMC4419806; doi:10.1093/gbe/evv060)
Supplement: Supplementary Data [file supp_evv060_Supplementary_Figres_S1-6.pdf]

A rpl19-atpA

Rpl19

```
ATGAAGATATACTCGCATAATCTTATACGTAAAATTATTGAAAGACGCTTGAAACTAAAT
 M  K  M  Y  S  H  N  L  M  R  K  I  I  E  R  R  L  K  L  N
ATAGATCTATTCAAAGTAGGAGATACAATTCAAGTATATGGTAAACTTCAAGAAAGCGAA
 M  D  L  F  K  V  G  D  T  I  Q  V  Y  G  K  L  Q  E  S  E
AGACAGCGCATTCAATTTTATCTAGGGATCTTGCAAATAAAAAAAGGAATTGGTAGAACA
 R  Q  R  I  Q  F  Y  L  G  I  L  Q  M  K  K  G  I  G  R  T
ACTATAAGAATTGTTCTGAGTGAATTTAATGGTGAAATCAAAATAAAATGTACATTTTAT
 T  M  R  I  V  L  S  E  F  N  G  E  I  K  M  K  C  T  F  Y
CTTAATTCCAAATTGATTGATTATATAGTAATTTTACACAAGCCAATCTATATATATTTT
 L  N  S  K  L  I  D  Y  M  V  I  L  H  K  P  I  Y  M  Y  F
TTGTTACCAAAAGCAGCTCTAATTTTTAAATATAAATTTTTTACTATGACAAAAATTCAA
 L  L  P  K  A  A  L  I  F  K  Y  K  F  F  T  M  T  K  I  Q
```

AtpA

```
CCCGAAGAAATTACTCGCATTATCAAAAGCCAACTACTTAGATCTAGTAAGAGCAAAAAT
 P  E  E  I  T  R  I  I  K  S  Q  L  L  R  S  S  K  S  K  N
TCGTTTATTCATGTGGGAACAGTACTTCAAATTGGCGATGGAATTGTACGTATTTATGGA
 S  F  I  H  V  G  T  V  L  Q  I  G  D  G  I  V  R  I  Y  G
CTAAATAAAGCAATAGCTGGCGAACTTTTGCGATTTGAAGTTGAAGGATTTACCGTCGGA
 L  N  K  A  M  A  G  E  L  L  R  F  E  V  E  G  F  T  V  G
ATTGCTTTGAATCTAGAATACAGCAACGTTGGTGCAGTTCTCATAGGAGAAAGCCGTAAT
 I  A  L  N  L  E  Y  S  N  V  G  A  V  L  M  G  E  S  R  N
ATTCGTGAAGGTACTAAAGTATGGGCAACAGGAAAAATTGCGCAAGTTCCCGTAGGAAAA
 I  R  E  G  T  K  V  W  A  T  G  K  I  A  Q  V  P  V  G  K
GAACTTTTGGGTCGAATTGTAAATTCGCTGGCAGCCCCTGTCGATGGTCTAGGCGATATT
 E  L  L  G  R  I  V  N  S  L  A  A  P  V  D  G  L  G  D  I
TATTCTTGTGCGATCCATCTTGTAGAGGCACCTGCGCCTGGTATTATTGACCGTCGATCC
 Y  S  C  A  I  H  L  V  E  A  P  A  P  G  I  I  D  R  R  S
GTTTTTGAACCGTTGCAAACTGGCATCCTTTCTATCGATGCTATAATTCCAATTGGTCGT
 V  F  E  P  L  Q  T  G  I  L  S  I  D  A  M  I  P  I  G  R
GGACAACGCGAATTGGTAATTGGTGACCGTCAAACAGGGAAAACTGCTGTTGCAATTGAT
 G  Q  R  E  L  V  I  G  D  R  Q  T  G  K  T  A  V  A  I  D
GCGATTCTTAACCAAACAGGAGAAAGTGTAAAATGCGTTTACGTTGCGATTGGTCAAAAA
 A  I  L  N  Q  T  G  E  S  V  K  C  V  Y  V  A  I  G  Q  K
GCATCTTCGGTTGCTCAAGTTGTGAATAATCTAAAAGAACGTGGAGCAATAGACTATACA
 A  S  S  V  A  Q  V  V  N  N  L  K  E  R  G  A  M  D  Y  T
GTCATTGTCGCAGCTACAGCTGATAGCCCAGCTACACTTCAATATTTGGCTCCATATACA
 V  I  V  A  A  T  A  D  S  P  A  T  L  Q  Y  L  A  P  Y  T
GGAGCATCGATTGCAGAACACTTTATGTATGGGGGGTCGCATACTCTTATAGTTTACGAC
 G  A  S  I  A  E  H  F  M  Y  G  G  S  H  T  L  M  V  Y  D
GACTTGTCTAAACATGCTCAGGCGTATCGAGAAATATCTTTGTTGTTGCGCCGACCACCT
 D  L  S  K  H  A  Q  A  Y  R  E  M  S  L  L  L  R  R  P  P
GGACGTGAAGCATATCCTGGAGATATTTTTTATGTACATTCGCGATTGTTGGAACGTGCA
 G  R  E  A  Y  P  G  D  I  F  Y  V  H  S  R  L  L  E  R  A
GCTCGATTGTTTGACAAATTGGGTGGAGGCAGTATAACAGCTTTGCCTATTGTAGAAACT
 A  R  L  F  D  K  L  G  G  G  S  M  T  A  L  P  I  V  E  T
CAAGCAAGAGATGTATCTGCGTATATTCCAACAAATGTTATTTCGATCACCGATGGACAA
 Q  A  R  D  V  S  A  Y  I  P  T  N  V  I  S  I  T  D  G  Q
ATTTTTCTTTCGGCGGATCTATTTAATTCGGGTTTGCGTCCTGCTGTAGATGTAGGAATT
 I  F  L  S  A  D  L  F  N  S  G  L  R  P  A  V  D  V  G  I
TCTGTTTCTCGTGTCGGATCTGCTGCGCAGAAAAAGCCTATAAAGCAAGTTTCGGGAAAA
 S  V  S  R  V  G  S  A  A  Q  K  K  P  M  K  Q  V  S  G  K
CTTAAGCTAGAATTGGCACAATTTGCTGAACTAGAAGCCTTTGCACAATTCGCTTCTGAC
 L  K  L  E  L  A  Q  F  A  E  L  E  A  F  A  Q  F  A  S  D
CTAGACGCTGCAACTCAAAGCCAACTTGCGCGTGGACGACGCCTTCGAGAACTGCTGAAA
 L  D  A  A  T  Q  S  Q  L  A  R  G  R  R  L  R  E  L  L  K
CAACAACAAAATTCCCCATTTCCTGTACAAAATATGGTGTTCATAATTTTTGCAAGAACG
 Q  Q  Q  N  S  P  F  P  V  Q  N  M  V  F  M  I  F  A  R  T
AATGGCTTTCTCGATAAGTGTGAAATGTCGGAAGTTCAAGGCGAGCTTCAGCGATTTGTG
 N  G  F  L  D  K  C  E  M  S  E  V  Q  G  E  L  Q  R  F  V
CGAAAAGCTATAAATAAGTATAGCACAGAAGATTTTAGTACAAACCCACTTCCTTCTGAT
 R  K  A  M  N  K  Y  S  T  E  D  F  S  T  N  P  L  P  S  D
TTTGGAGAAAAAATAAAAGAAATTATACTTGCTGACTAG
 F  G  E  K  M  K  E  I  M  L  A  D  *
```

B psbK-rpl20

PsbK

```
ATGATTTATTCTTTGGCAAAACTTCCTGAAGCATACTCGCTAGTGAAATCAATTGTAGAT
 M  I  Y  S  L  A  K  L  P  E  A  Y  S  L  V  K  S  I  V  D
GTGCTACCTATTGTTCCTGTATTTTTTCTTCTTCTTGCTTTTGTATGGCAAGCTTCGGTA
 V  L  P  I  V  P  V  F  F  L  L  L  A  F  V  W  Q  A  S  V
AGTTTTCGTTTAATTATATATTATAAAAAAATATTTTTCCTTATGAGACGAGTGAAACGC
 S  F  R  L  I  M  Y  Y  K  K  M  F  F  L  M  R  R  V  K  R
```

Rpl20

```
GAAATCAATGCACAAATTCGACGACAAAAAATCCTTTTGTTGAGAAAAAGAGCAATAGAA
 E  I  N  A  Q  I  R  R  Q  K  I  L  L  L  R  K  R  A  M  E
TCTAATTCTCGTTCGTTTCGAATAGCGAAACAACAACTTATAAAATCTATAAATTTTTCG
 S  N  S  R  S  F  R  M  A  K  Q  Q  L  M  K  S  M  N  F  S
TATTGTAGTCAACAAAATCGAAAACGCTATTTTCGATCCCTCTGGATTATTCGACTTAAC
 Y  C  S  Q  Q  N  R  K  R  Y  F  R  S  L  W  I  I  R  L  N
AGAGCCCTAAAAACGTGCACAATTTCTGATCGCAATATTATATTTAAAAATAGATTGATT
 R  A  L  K  T  C  T  I  S  D  R  N  I  M  F  K  N  R  L  I
GAGAAGGATACGTTTTTTTTCTCGGATAAATGTAATTATCTTACATTCATTTACAGCATC
 E  K  D  T  F  F  F  S  D  K  C  N  Y  L  T  F  I  Y  S  I
CGACAAAGAAAGTGCATTCTAAATCGAAAAGTAATGGCACAAATAGTACTACTCGACCCA
 R  Q  R  K  C  I  L  N  R  K  V  M  A  Q  M  V  L  L  D  P
CCATTTTTTTATAAATTGGTAGATGAGGCTCTATTTATATTGTAG
 P  F  F  Y  K  L  V  D  E  A  L  F  M  L  *
```

Figure S1 (Kamikawa et al.)

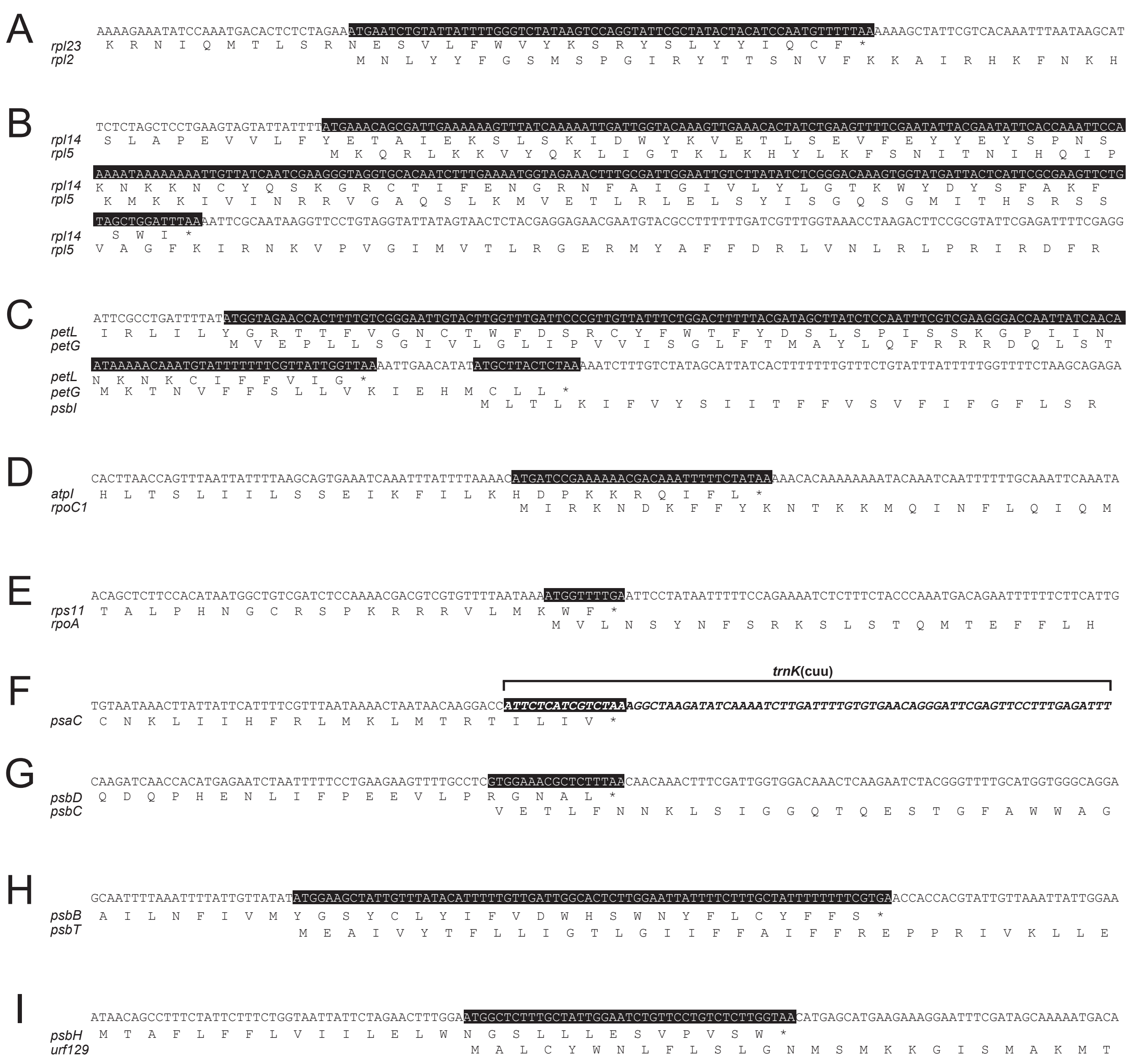

Fig. S2 (Kamikawa et al.)

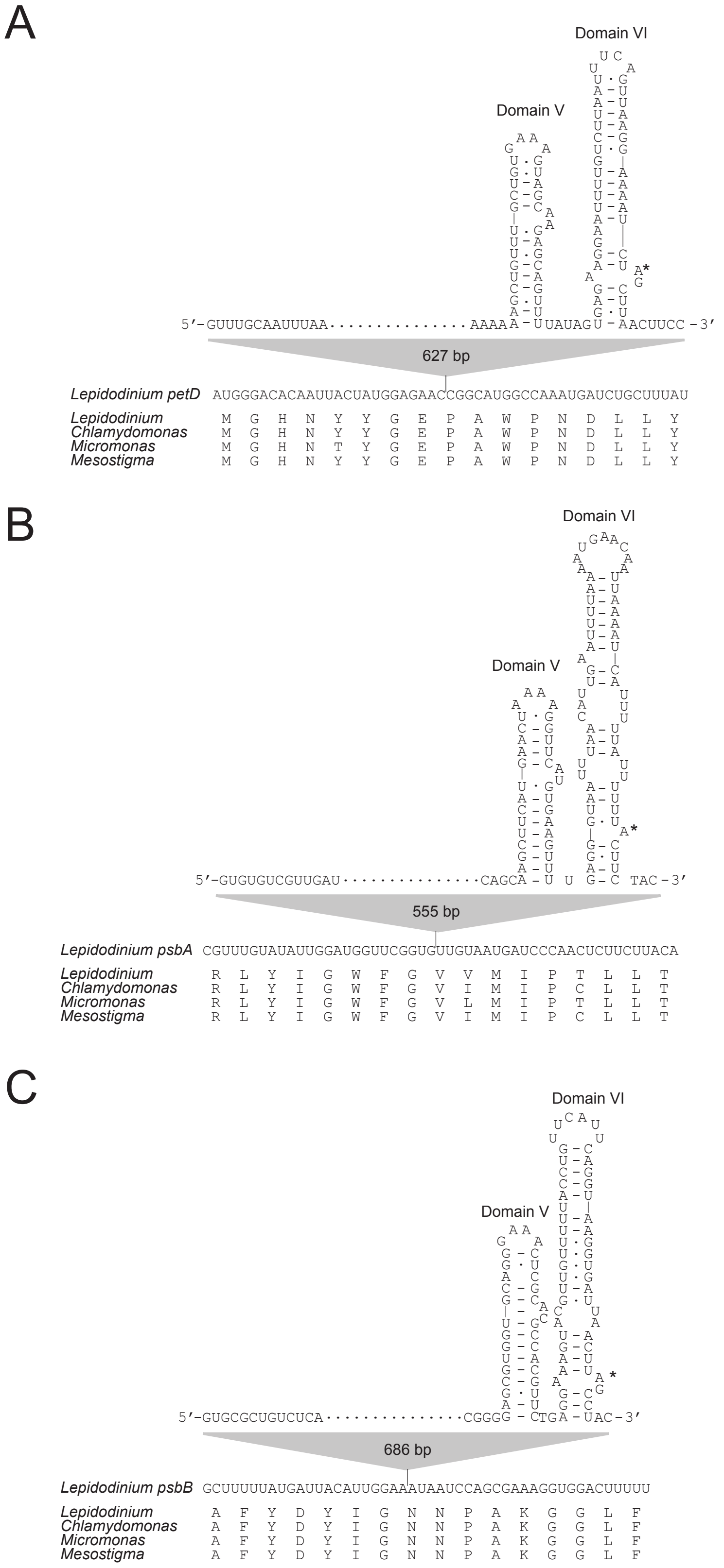

Fig. S3 (Kamikawa et al.)

```
ycf3   ATGTCTCGATCTCCACGGAATGACAATTTCATTGACAAAACATTTACAATTATCGCAAAT
ψycf3  ATGTCTGAATCTCCAAAGAA------------TGACAAAACATTTATAATTATCGCAAAT
       ******  *******  ***            ************** *************

ycf3   TTAATTTTGAAAGGCATTCCTACTCCAAAAACAGCTAAAATTGCTTTTGCATTTTATCGT
ψycf3  TTAATTTTGAAAGGCATTCCTATTCAAAAAACAGATAAAATAGTTTATGCATTTTATCCT
       ********************** ** ******** ****** * ** *********** *

ycf3   GATGGACTTGCTGCCCAATCGGAGGGAGAATATGCAGAAGCTCTTAAAAACTATTTTAAG
ψycf3  GATGGATTCACCGCACAATCGAAGAACGAATATGTATAA---------------------
       ****** *  * ** ****** **   ******* * **

ycf3   GCACTTCGCCTCGAAACAGACCCGTTTGATCGGAGTTACATTCTCTATAATATTAGATTG
ψycf3  ------------------------------------------------------------

ycf3   ATTCATACTTCGAATGGACGTTATTGTCGAGCCCTTGAGTATTATTTTGCAGCTTTGGAT
ψycf3  ------------------------------------------------------------

ycf3   CGAAATCCGGGACTAGTGCAAGCTCTTAATAATATCAGAATCTTGTACCATTCGCGCGGA
ψycf3  ------------------------------------------------------------

ycf3   GAAGAAGCTGTAAAAAAAAATAAATACGAAGTGTCTAAGCTTCTATTTTCTCGAAGCGCC
ψycf3  ------------------------------------------------------------

ycf3   GAATATTGGAAAGAAGCAATTCGACTCACACCTTCTAATTACATTGAAGCTCAAAATTGG
ψycf3  ------------------------------------------------------------

ycf3   ATTTCGATAAATTCTTCCAATTACAAACATAAATAA
ψycf3  ------------------------------------
```

Figure S4 (Kamikawa et al.)

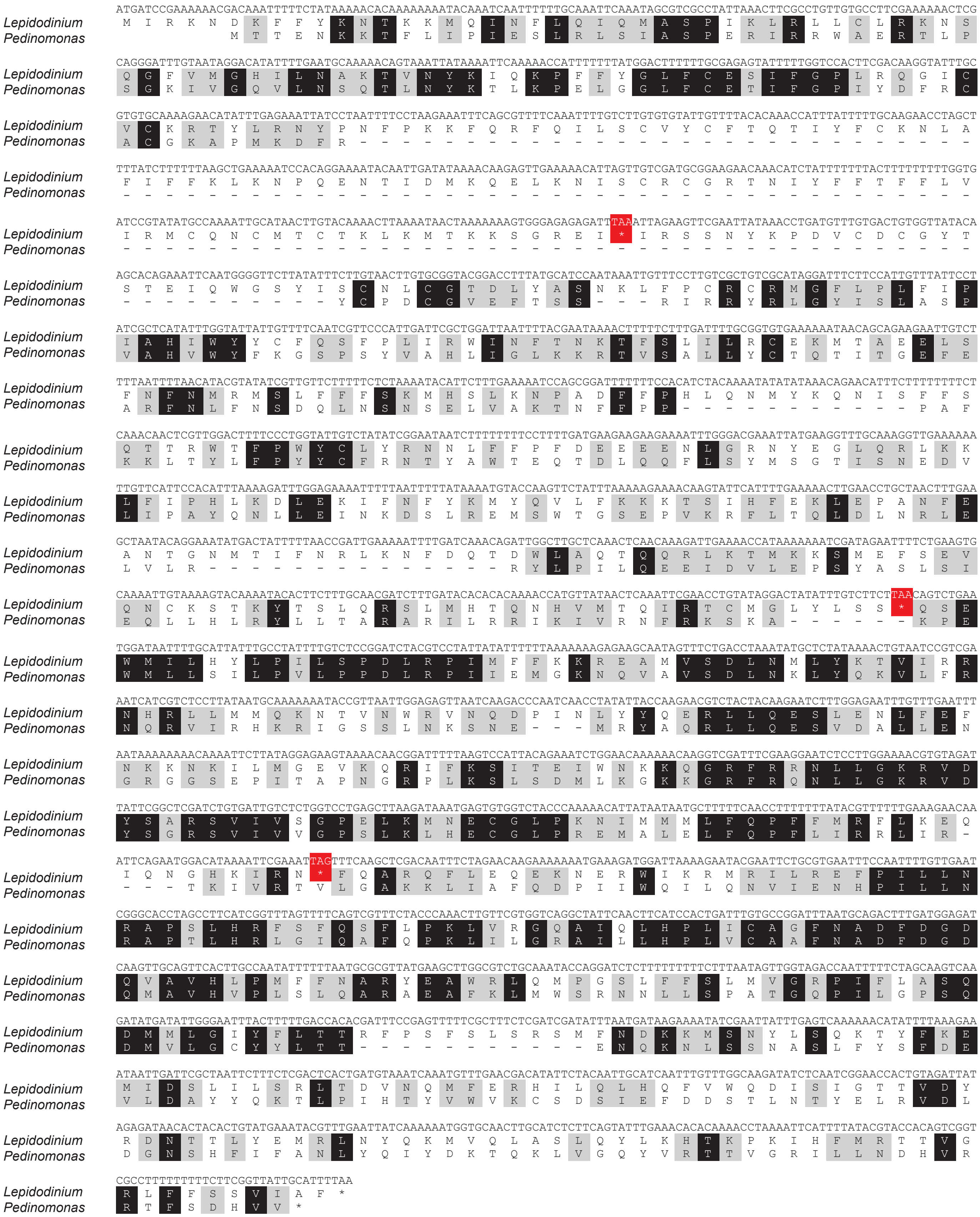

Fig. S5 (Kamikawa et al.)

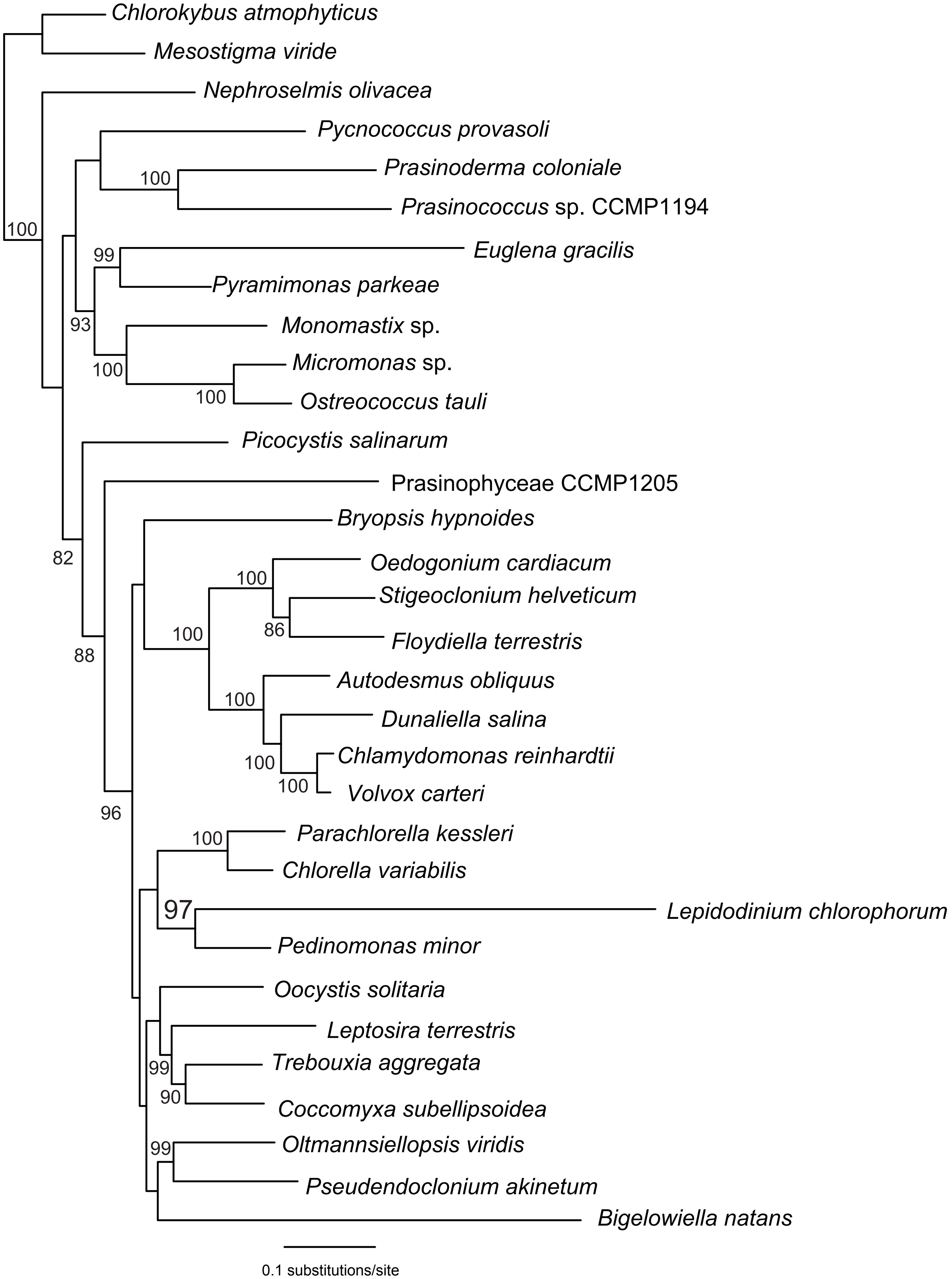

Fig. S6 (Kamikawa et al.)
