# Supplementary material for: Plastid Genome-Based Phylogeny Pinpointed the Origin of the Green-Colored Plastid in the Dinoflagellate Lepidodinium chlorophorum
Source: Genome Biol Evol. 2015 Apr 2;7(4):1133–40. doi: 10.1093/gbe/evv060 (PMC4419806; doi:10.1093/gbe/evv060)
Supplement: Supplementary Data [file supp_evv060_Supplementary_Tables_S1_S2.pdf]

Table S1. Protein data used for the phylogenomic analyses

	atpA		atpB		atpE		atpF	
Taxa	aa	missing (%)	aa	missing (%)	aa	missing (%)	aa	missing (%)
Bigelowiella natans	437	0	446	0.2	118	0	70	0
Bryopsis hypnoides	437	0	447	0	118	0	70	0
Chlamydomonas reinhardtii	437	0	447	0	118	0	70	0
Chlorella variabilis	437	0	447	0	118	0	70	0
Chlorokybus atmophyticus	437	0	447	0	118	0	70	0
Coccomyxa subellipsoidea	437	0	447	0	118	0	70	0
Dunaliella salina	437	0	447	0	118	0	70	0
Euglena gracilis	437	0	447	0	118	0	70	0
Floydiella terrestris	437	0	447	0	118	0	70	0
Lepidodinium chlorophorum	437	0	447	0	118	0	70	0
Leptosira terrestris	437	0	447	0	118	0	70	0
Mesostigma viride	437	0	447	0	118	0	70	0
Micromonas sp. RCC299	437	0	447*[1]	0	118	0	70	0
Monomastix sp.	437	0	447	0	118	0	70	0
Nephroselmis olivacea	437	0	447	0	118	0	70	0
Oedogonium cardiacum	437	0	447	0	118	0	70	0
Oltmannsiellopsis viridis	437	0	447	0	118	0	70	0
Oocystis solitaria	437	0	447	0	118	0	70	0
Ostreococcus tauli	437	0	447	0	118	0	70	0
Parachlorella kessleri	437	0	447	0	118	0	70	0
Pedinomonas minor	437	0	447	0	118	0	70	0
Picocystis salinarum	437	0	447	0	118	0	70	0
Prasinococcus sp. CCMP1194	437	0	447	0	118	0	70	0
Prasinoderma coloniale	437	0	447	0	118	0	70	0
Prasinophyceae sp. CCMP1205	437	0	447	0	118	0	70	0
Pseudendoclonium akinetum	437	0	447	0	118	0	70	0
Pycnococcus provasoli	437	0	447	0	118	0	70	0
Pyramimonas parkeae	437	0	447	0	118	0	70	0
Autodesmus obliquus	437	0	447	0	118	0	70	0
Stigeoclonium helveticum	437	0	447	0	118	0	70	0
Trebouxia aggregata	437	0	447	0	118	0	70	0
Volvox carteri	437	0	447	0	118	0	70	0

*1: Not annotated in NC_012575
*2: Not annotated in GQ892829

Table S1. Continued.

	atpH		atpI		ClpP		petA	
Taxa	aa	missing (%)	aa	missing (%)	aa	missing (%)	aa	missing (%)
Bigelowiella natans	79	1.3	219	0	144	0	274	0.7
Bryopsis hypnoides	80	0	219	0	144	0	276	0
Chlamydomonas reinhardtii	80	0	219	0	144	0	276	0
Chlorella variabilis	80	0	219	0	144	0	276	0
Chlorokybus atmophyticus	80	0	219	0	144	0	276	0
Coccomyxa subellipsoidea	57	28.8	219	0	144	0	276	0
Dunaliella salina	80	0	219	0	144	0	276	0
Euglena gracilis	79	1.3	219	0	0	100	0	100
Floydiella terrestris	80	0	219	0	144	0	0	100
Lepidodinium chlorophorum	80	0	219	0	144	0	274	0.7
Leptosira terrestris	80	0	219	0	144	0	276	0
Mesostigma viride	80	0	219	0	144	0	275	0.4
Micromonas sp. RCC299	80	0	218	0.5	144	0	276	0
Monomastix sp.	80	0	219	0	144	0	276	0
Nephroselmis olivacea	80	0	218	0.5	144	0	276	0
Oedogonium cardiacum	80	0	219	0	144	0	0	100
Oltmannsiellopsis viridis	80	0	219	0	144	0	276	0
Oocystis solitaria	80	0	219	0	144	0	276	0
Ostreococcus tauli	80	0	218	0.5	144	0	276	0
Parachlorella kessleri	80	0	219	0	144	0	276	0
Pedinomonas minor	80	0	219	0	144	0	276	0
Picocystis salinarum	80	0	219	0	144	0	275	0.4
Prasinococcus sp. CCMP1194	80	0	218	0.5	144	0	276	0
Prasinoderma coloniale	79	1.3	219	0	144	0	276	0
Prasinophyceae sp. CCMP120[illegible]	80	0	219	0	144	0	271	1.8
Pseudendoclonium akinetum	80	0	219	0	144	0	276	0
Pycnococcus provasoli	80	0	219	0	144	0	276	0
Pyramimonas parkeae	80	0	219	0	144	0	276	0
Autodesmus obliquus	80	0	219	0	144	0	276	0
Stigeoclonium helveticum	80	0	219	0	144	0	0	100
Trebouxia aggregata	80	0	219	0	144	0	276	0
Volvox carteri	80	0	219	0	144	0	276	0

Table S1. Continued.

	petB		petD		petG		petL	
Taxa	aa	missing (%)	aa	missing (%)	aa	missing (%)	aa	missing (%)
Bigelowiella natans	215	0	160	0	31	0	0	100
Bryopsis hypnoides	215	0	160	0	31	0	31	0
Chlamydomonas reinhardtii	215	0	160	0	31	0	31	0
Chlorella variabilis	215	0	160	0	31	0	31	0
Chlorokybus atmophyticus	215	0	160	0	31	0	31	0
Coccomyxa subellipsoidea	215	0	160	0	31	0	31	0
Dunaliella salina	215	0	160	0	31	0	31	0
Euglena gracilis	215	0	0	100	31	0	0	100
Floydiella terrestris	215	0	160	0	31	0	31	0
Lepidodinium chlorophorum	215	0	160	0	31	0	31	0
Leptosira terrestris	215	0	160	0	31	0	31	0
Mesostigma viride	215	0	160	0	31	0	31	0
Micromonas sp. RCC299	215	0	160	0	31	0	0	100
Monomastix sp.	215	0	160	0	31	0	0	100
Nephroselmis olivacea	215	0	160	0	31	0	31	0
Oedogonium cardiacum	215	0	160	0	31	0	31	0
Oltmannsiellopsis viridis	215	0	160	0	31	0	31	0
Oocystis solitaria	215	0	160	0	31	0	31	0
Ostreococcus tauli	215	0	0	100	31	0	0	100
Parachlorella kessleri	215	0	160	0	31	0	31	0
Pedinomonas minor	215	0	160	0	31	0	31	0
Picocystis salinarum	215	0	160	0	31	0	31	0
Prasinococcus sp. CCMP1194	215	0	160	0	31	0	0	100
Prasinoderma coloniale	215	0	160	0	31	0	0	100
Prasinophyceae sp. CCMP120[illegible]	215	0	160	0	31	0	31	0
Pseudendoclonium akinetum	215	0	160	0	31	0	31	0
Pycnococcus provasoli	215	0	160	0	31	0	31	0
Pyramimonas parkeae	215	0	0	100	31	0	0	100
Autodesmus obliquus	215	0	160	0	31	0	31	0
Stigeoclonium helveticum	215	0	160	0	31	0	31	0
Trebouxia aggregata	215	0	160	0	0	100	31	0
Volvox carteri	215	0	160	0	31	0	31	0

Table S1. Continued.

	psaA		psaB		psaC		psaI	
Taxa	aa	missing (%)	aa	missing (%)	aa	missing (%)	aa	missing (%)
Bigelowiella natans	749	0	708	3.0	79	2.5	0	100
Bryopsis hypnoides	749	0	730	0	81	0	29*2	0
Chlamydomonas reinhardtii	749	0	730	0	81	0	0	100
Chlorella variabilis	680	9.2	730	0	81	0	29	0
Chlorokybus atmophyticus	748	0.1	730	0	81	0	29	0
Coccomyxa subellipsoidea	749	0	730	0	81	0	29	0
Dunaliella salina	749	0	730	0	81	0	0	100
Euglena gracilis	749	0	730	0	81	0	0	100
Floydiella terrestris	749	0	730	0	81	0	0	100
Lepidodinium chlorophorum	749	0	730	0	81	0	29	0
Leptosira terrestris	749	0	730	0	81	0	29	0
Mesostigma viride	748	0.1	730	0	81	0	29	0
Micromonas sp. RCC299	749	0	730	0	81	0	29	0
Monomastix sp.	747	0.3	730	0	0	100	29	0
Nephroselmis olivacea	749	0	730	0	81	0	29	0
Oedogonium cardiacum	749	0	730	0	81	0	0	100
Oltmannsiellopsis viridis	749	0	730	0	81	0	29	0
Oocystis solitaria	749	0	730	0	81	0	29	0
Ostreococcus tauli	749	0	729	0.1	81	0	29	0
Parachlorella kessleri	749	0	730	0	81	0	29	0
Pedinomonas minor	749	0	730	0	81	0	29	0
Picocystis salinarum	749	0	730	0	81	0	29	0
Prasinococcus sp. CCMP1194	749	0	730	0	81	0	29	0
Prasinoderma coloniale	749	0	730	0	81	0	29	0
Prasinophyceae sp. CCMP120[illegible]	748	0.1	730	0	81	0	0	100
Pseudendoclonium akinetum	749	0	730	0	81	0	29	0
Pycnococcus provasoli	749	0	730	0	81	0	29	0
Pyramimonas parkeae	749	0	730	0	81	0	29	0
Autodesmus obliquus	749	0	730	0	81	0	0	100
Stigeoclonium helveticum	749	0	730	0	81	0	0	100
Trebouxia aggregata	749	0	730	0	81	0	29	0
Volvox carteri	749	0	730	0	81	0	0	100

Table S1. Continued.

	psaJ		psbA		psbB		psbC	
Taxa	aa	missing (%)	aa	missing (%)	aa	missing (%)	aa	missing (%)
Bigelowiella natans	39	0	341	0	508	0	461	0
Bryopsis hypnoides	39	0	341	0	508	0	461	0
Chlamydomonas reinhardtii	39	0	341	0	508	0	461	0
Chlorella variabilis	39	0	341	0	479	5.7	461	0
Chlorokybus atmophyticus	39	0	341	0	508	0.0	461	0
Coccomyxa subellipsoidea	39	0	341	0	506	0.4	455	1.3
Dunaliella salina	39	0	341	0	508	0	461	0
Euglena gracilis	37	5.1	341	0	508	0	461	0
Floydiella terrestris	39	0	341	0	508	0	461	0
Lepidodinium chlorophorum	37	5.1	341	0	489	3.7	461	0
Leptosira terrestris	39	0	341	0	508	0	461	0
Mesostigma viride	39	0.0	341	0	508	0.0	461	0
Micromonas sp. RCC299	39	0	341	0	497	2.2	461	0
Monomastix sp.	39	0	341	0	508	0	461	0
Nephroselmis olivacea	39	0	341	0	508	0	461	0
Oedogonium cardiacum	39	0	341	0	508	0	461	0
Oltmannsiellopsis viridis	39	0	341	0	508	0	461	0
Oocystis solitaria	39	0	341	0	508	0	461	0
Ostreococcus tauli	39	0	341	0	443	12.8	461	0
Parachlorella kessleri	39	0	341	0	508	0	461	0
Pedinomonas minor	39	0	341	0	508	0	461	0
Picocystis salinarum	0	100	341	0	508	0.0	461	0
Prasinococcus sp. CCMP1194	39	0	341	0	508	0.0	461	0
Prasinoderma coloniale	39	0	341	0	508	0.0	461	0
Prasinophyceae sp. CCMP120[illegible]	39	0	341	0	508	0.0	461	0
Pseudendoclonium akinetum	39	0	341	0	508	0	461	0
Pycnococcus provasoli	0	100	341	0	508	0	461	0
Pyramimonas parkeae	39	0	341	0	508	0	461	0
Autodesmus obliquus	39	0	341	0	508	0	461	0
Stigeoclonium helveticum	39	0	341	0	507	0.2	461	0
Trebouxia aggregata	39	0	341	0	508	0	461	0
Volvox carteri	39	0	341	0	508	0	461	0

Table S1. Continued.

	psbD		psbE		psbF		psbH	
Taxa	aa	missing (%)	aa	missing (%)	aa	missing (%)	aa	missing (%)
Bigelowiella natans	352	0	70	7.9	34	0	59	1.7
Bryopsis hypnoides	352	0	76	0	34	0	60	0
Chlamydomonas reinhardtii	352	0	76	0	34	0	60	0
Chlorella variabilis	352	0	76	0	34	0	60	0
Chlorokybus atmophyticus	352	0	76	0	34	0	60	0
Coccomyxa subellipsoidea	352	0	76	0	34	0	60	0
Dunaliella salina	352	0	76	0	34	0	60	0
Euglena gracilis	342	2.8	76	0	34	0	56	6.7
Floydiella terrestris	352	0	76	0	34	0	60	0
Lepidodinium chlorophorum	352	0	76	0	34	0	60	0
Leptosira terrestris	352	0	76	0	34	0	60	0
Mesostigma viride	352	0	76	0	34	0	60	0
Micromonas sp. RCC299	352	0	76	0	34	0	60	0
Monomastix sp.	352	0	76	0	34	0	60	0
Nephroselmis olivacea	352	0	76	0	34	0	60	0
Oedogonium cardiacum	352	0	76	0	34	0	60	0
Oltmannsiellopsis viridis	352	0	76	0	34	0	60	0
Oocystis solitaria	352	0	76	0	34	0	60	0
Ostreococcus tauli	352	0	76	0	34	0	60	0
Parachlorella kessleri	352	0	76	0	34	0	60	0
Pedinomonas minor	352	0	76	0	34	0	60	0
Picocystis salinarum	352	0	76	0	34	0	60	0
Prasinococcus sp. CCMP1194	352	0	76	0	34	0	60	0
Prasinoderma coloniale	352	0	76	0	34	0	60	0
Prasinophyceae sp. CCMP120[illegible]	352	0	76	0	34	0	60	0
Pseudendoclonium akinetum	352	0	76	0	34	0	60	0
Pycnococcus provasoli	352	0	76	0	34	0	60	0
Pyramimonas parkeae	352	0	76	0	34	0	60	0
Autodesmus obliquus	352	0	76	0	34	0	60	0
Stigeoclonium helveticum	352	0	76	0	34	0	60	0
Trebouxia aggregata	352	0	76	0	34	0	60	0
Volvox carteri	352	0	76	0	34	0	60	0

Table S1. Continued.

	psbI		psbJ		psbK		psbL	
Taxa	aa	missing (%)	aa	missing (%)	aa	missing (%)	aa	missing (%)
Bigelowiella natans	32	0	42	0	39	0	34	0
Bryopsis hypnoides	32	0	42	0	39	0	34	0
Chlamydomonas reinhardtii	32	0	42	0	39	0	34	0
Chlorella variabilis	32	0	42	0	39	0	34	0
Chlorokybus atmophyticus	32	0	42	0	39	0	34	0
Coccomyxa subellipsoidea	32	0	42	0	39	0	34	0
Dunaliella salina	32	0	42	0	39	0	34	0
Euglena gracilis	32	0	42	0	39	0	34	0
Floydiella terrestris	32	0	42	0	39	0	34	0
Lepidodinium chlorophorum	32	0	42	0	39	0	34	0
Leptosira terrestris	32	0	42	0	39	0	34	0
Mesostigma viride	32	0	42	0	39	0	34	0
Micromonas sp. RCC299	32	0	41	2.4	39	0	34	0
Monomastix sp.	32	0	42	0	39	0	34	0
Nephroselmis olivacea	32	0	42	0	39	0	34	0
Oedogonium cardiacum	32	0	42	0	39	0	34	0
Oltmannsiellopsis viridis	32	0	42	0	39	0	34	0
Oocystis solitaria	32	0	42	0	39	0	34	0
Ostreococcus tauli	32	0	41	2.4	39	0	34	0
Parachlorella kessleri	32	0	42	0	39	0	34	0
Pedinomonas minor	32	0	42	0	39	0	34	0
Picocystis salinarum	32	0	42	0	39	0	34	0
Prasinococcus sp. CCMP1194	32	0	42	0	39	0	34	0
Prasinoderma coloniale	32	0	42	0	39	0	34	0
Prasinophyceae sp. CCMP120[illegible]	32	0	42	0	39	0	34	0
Pseudendoclonium akinetum	32	0	42	0	39	0	34	0
Pycnococcus provasoli	32	0	42	0	39	0	34	0
Pyramimonas parkeae	32	0	42	0	39	0	34	0
Autodesmus obliquus	32	0	42	0	39	0	34	0
Stigeoclonium helveticum	32	0	42	0	39	0	34	0
Trebouxia aggregata	0	100	42	0	39	0	34	0
Volvox carteri	32	0	42	0	39	0	17	50.0

Table S1. Continued.

	psbM		psbN		psbT		psbZ	
Taxa	aa	missing (%)	aa	missing (%)	aa	missing (%)	aa	missing (%)
Bigelowiella natans	31	0	44	0	31	0	0	100
Bryopsis hypnoides	31	0	44	0	31	0	62	0
Chlamydomonas reinhardtii	31	0	44	0	31	0	62	0
Chlorella variabilis	31	0	44	0	31	0	62	0
Chlorokybus atmophyticus	31	0	43	2.3	31	0	62	0
Coccomyxa subellipsoidea	31	0	44	0	31	0	62	0
Dunaliella salina	31	0	44	0	31	0	62	0
Euglena gracilis	0	100	44	0	31	0	62	0
Floydiella terrestris	31	0	44	0	31	0	62	0
Lepidodinium chlorophorum	31	0	44	0	31	0	62	0
Leptosira terrestris	31	0	44	0	31	0	62	0
Mesostigma viride	31	0	43	2.3	31	0	62	0
Micromonas sp. RCC299	0	100	44	0	31	0	62	0
Monomastix sp.	31	0	44	0	31	0	62	0
Nephroselmis olivacea	31	0	44	0	31	0	62	0
Oedogonium cardiacum	31	0	44	0	31	0	62	0
Oltmannsiellopsis viridis	31	0	44	0	31	0	62	0
Oocystis solitaria	31	0	44	0	31	0	62	0
Ostreococcus tauli	0	100	44	0	31	0	62	0
Parachlorella kessleri	31	0	44	0	31	0	62	0
Pedinomonas minor	31	0	44	0	31	0	62	0
Picocystis salinarum	31	0	44	0	31	0	62	0
Prasinococcus sp. CCMP1194	0	100	43	2.3	31	0	62	0
Prasinoderma coloniale	0	100	43	2.3	31	0	62	0
Prasinophyceae sp. CCMP120[illegible]	31	0	44	0	31	0	62	0
Pseudendoclonium akinetum	31	0	44	0	31	0	62	0
Pycnococcus provasoli	0	100	44	0	31	0	62	0
Pyramimonas parkeae	0	100	44	0	31	0	62	0
Autodesmus obliquus	31	0	44	0	31	0	62	0
Stigeoclonium helveticum	30	3.2	44	0	31	0	62	0
Trebouxia aggregata	31	0	44	0	31	0	62	0
Volvox carteri	30	3.2	44	0	31	0	62	0

Table S1. Continued.

	rbcL		rpl2		rpl5		rpl14	
Taxa	aa	missing (%)	aa	missing (%	aa	missing (%	aa	missing (%)
Bigelowiella natans	466	1.9	192	24.1	154	0	113	0
Bryopsis hypnoides	475	0	253	0	152	1.3	113	0
Chlamydomonas reinhardtii	475	0	253	0	154	0	113	0
Chlorella variabilis	475	0	253	0	154	0	113	0
Chlorokybus atmophyticus	475	0	253	0	154	0	113	0
Coccomyxa subellipsoidea	475	0	253	0	154	0	113	0
Dunaliella salina	475	0	253	0	154	0	113	0
Euglena gracilis	475	0	253	0	154	0	113	0
Floydiella terrestris	475	0	253	0	154	0	113	0
Lepidodinium chlorophorum	475	0	253	0	152	1.3	113	0
Leptosira terrestris	475	0	253	0	154	0	113	0
Mesostigma viride	475	0	252	0.4	154	0	113	0
Micromonas sp. RCC299	475	0	253	0	154	0	113	0
Monomastix sp.	475	0	253	0	151	1.9	113	0
Nephroselmis olivacea	475	0	253	0	154	0	113	0
Oedogonium cardiacum	475	0	253	0	154	0	113	0
Oltmannsiellopsis viridis	474	0.2	253	0	154	0	113	0
Oocystis solitaria	475	0	253	0	154	0	113	0
Ostreococcus tauli	475	0	253	0	154	0	113	0
Parachlorella kessleri	475	0	253	0	154	0	113	0
Pedinomonas minor	475	0	253	0	154	0	113	0
Picocystis salinarum	475	0	253	0	154	0	113	0
Prasinococcus sp. CCMP1194	475	0	253	0	154	0	113	0
Prasinoderma coloniale	475	0	252	0.4	154	0	113	0
Prasinophyceae sp. CCMP120[illegible]	475	0	253	0	154	0	113	0
Pseudendoclonium akinetum	475	0	253	0	154	0	113	0
Pycnococcus provasoli	475	0	253	0	154	0	113	0
Pyramimonas parkeae	475	0	253	0	154	0	113	0
Autodesmus obliquus	475	0	253	0	154	0	113	0
Stigeoclonium helveticum	475	0	253	0	154	0	113	0
Trebouxia aggregata	475	0	253	0	154	0	113	0
Volvox carteri	475	0	253	0	154	0	113	0

Table S1. Continued.

	rpl16		rpl20		rpl23		rpl36	
Taxa	aa	missing (%)	aa	missing (%)	aa	missing (%)	aa	missing (%)
Bigelowiella natans	131	0	101	0	36	0	37	0
Bryopsis hypnoides	131	0	101	0	36	0	36	2.7
Chlamydomonas reinhardtii	131	0	101	0	36	0	37	0
Chlorella variabilis	131	0	101	0	36	0	37	0
Chlorokybus atmophyticus	131	0	101	0	36	0	37	0
Coccomyxa subellipsoidea	131	0	101	0	36	0	37	0
Dunaliella salina	131	0	101	0	36	0	37	0
Euglena gracilis	131	0	101	0	32	11.1	37	0
Floydiella terrestris	131	0	101	0	36	0	37	0
Lepidodinium chlorophorum	131	0	101	0	36	0	37	0
Leptosira terrestris	131	0	101	0	36	0	37	0
Mesostigma viride	131	0	101	0	36	0	37	0
Micromonas sp. RCC299	131	0	101	0	36	0	0	100
Monomastix sp.	131	0	101	0	36	0	37	0
Nephroselmis olivacea	131	0	101	0	36	0	37	0
Oedogonium cardiacum	131	0	101	0	36	0	37	0
Oltmannsiellopsis viridis	131	0	101	0	36	0	37	0
Oocystis solitaria	131	0	101	0	36	0	37	0
Ostreococcus tauli	131	0	101	0	36	0	37	0
Parachlorella kessleri	131	0	101	0	36	0	37	0
Pedinomonas minor	131	0	101	0	36	0	37	0
Picocystis salinarum	131	0	101	0	36	0	37	0
Prasinococcus sp. CCMP1194	131	0	101	0	36	0	37	0
Prasinoderma coloniale	131	0	101	0	36	0	37	0
Prasinophyceae sp. CCMP120[illegible]	131	0	101	0	36	0	37	0
Pseudendoclonium akinetum	131	0	101	0	36	0	37	0
Pycnococcus provasoli	131	0	101	0	36	0	37	0
Pyramimonas parkeae	131	0	101	0	36	0	37	0
Autodesmus obliquus	131	0	101	0	36	0	37	0
Stigeoclonium helveticum	131	0	101	0	36	0	37	0
Trebouxia aggregata	131	0	101	0	36	0	37	0
Volvox carteri	131	0	101	0	36	0	37	0

Table S1. Continued.

	rps2		rps3		rps4		rps7	
Taxa	aa	missing (%)	aa	missing (%)	aa	missing (%)	aa	missing (%)
Bigelowiella natans	174	0	115	0	108	12.2	128	0
Bryopsis hypnoides	174	0	115	0	123	0	128	0
Chlamydomonas reinhardtii	174	0	115	0	123	0	128	0
Chlorella variabilis	174	0	115	0	123	0	128	0
Chlorokybus atmophyticus	174	0	115	0	115	6.5	128	0
Coccomyxa subellipsoidea	174	0	115	0	123	0	128	0
Dunaliella salina	174	0	115	0	123	0	128	0
Euglena gracilis	173	0.6	115	0	108	12.2	128	0
Floydiella terrestris	174	0	115	0	123	0	128	0
Lepidodinium chlorophorum	173	0.6	76	33.9	109	11.4	128	0
Leptosira terrestris	174	0	115	0	123	0	128	0
Mesostigma viride	174	0	115	0	114	7.3	128	0
Micromonas sp. RCC299	174	0	115	0	123	0	128	0
Monomastix sp.	174	0	115	0	123	0	128	0
Nephroselmis olivacea	174	0	115	0	123	0	128	0
Oedogonium cardiacum	174	0	115	0	123	0	128	0
Oltmannsiellopsis viridis	174	0	115	0	123	0	128	0
Oocystis solitaria	174	0	115	0	123	0	128	0
Ostreococcus tauli	174	0	115	0	123	0	128	0
Parachlorella kessleri	174	0	115	0	123	0	128	0
Pedinomonas minor	174	0	115	0	123	0	128	0
Picocystis salinarum	174	0	115	0	123	0	128	0
Prasinococcus sp. CCMP1194	174	0	115	0	115	6.5	128	0
Prasinoderma coloniale	174	0	115	0	123	0	128	0
Prasinophyceae sp. CCMP120[illegible]	174	0	111	3.5	115	6.5	128	0
Pseudendoclonium akinetum	174	0	115	0	123	0	128	0
Pycnococcus provasoli	174	0	115	0	123	0	128	0
Pyramimonas parkeae	174	0	115	0	123	0	128	0
Autodesmus obliquus	92	47.1	115	0	123	0	128	0
Stigeoclonium helveticum	174	0	115	0	123	0	128	0
Trebouxia aggregata	174	0	115	0	109	11.4	128	0
Volvox carteri	174	0	115	0	123	0	128	0

Table S1. Continued.

	rps9		rps11		rps12		rps14	
Taxa	aa	missing (%)	aa	missing (%)	aa	missing (%)	aa	missing (%)
Bigelowiella natans	0	100	120	0	119	0	100	0
Bryopsis hypnoides	88	0	120	0	119	0	100	0
Chlamydomonas reinhardtii	88	0	120	0	119	0	100	0
Chlorella variabilis	88	0	120	0	119	0	100	0
Chlorokybus atmophyticus	88	0	120	0	119	0	100	0
Coccomyxa subellipsoidea	88	0	120	0	119	0	100	0
Dunaliella salina	88	0	120	0	119	0	100	0
Euglena gracilis	88	0	120	0	119	0	100	0
Floydiella terrestris	88	0	120	0	119	0	100	0
Lepidodinium chlorophorum	88	0	119	0.8	119	0	100	0
Leptosira terrestris	88	0	120	0	119	0	100	0
Mesostigma viride	88	0	120	0	119	0	100	0
Micromonas sp. RCC299	88	0	120	0	119	0	100	0
Monomastix sp.	0	100	120	0	119	0	99	1
Nephroselmis olivacea	88	0	120	0	119	0	100	0
Oedogonium cardiacum	88	0	120	0	119	0	100	0
Oltmannsiellopsis viridis	88	0	120	0	119	0	100	0
Oocystis solitaria	88	0	120	0	119	0	100	0
Ostreococcus tauli	88	0	120	0	119	0	100	0
Parachlorella kessleri	88	0	120	0	119	0	100	0
Pedinomonas minor	88	0	120	0	119	0	100	0
Picocystis salinarum	88	0	120	0	119	0	100	0
Prasinococcus sp. CCMP1194	88	0	120	0	119	0	98	2
Prasinoderma coloniale	88	0	120	0	119	0	100	0
Prasinophyceae sp. CCMP120[illegible]	88	0	118	1.7	119	0	100	0
Pseudendoclonium akinetum	88	0	120	0	119	0	100	0
Pycnococcus provasoli	0	100	120	0	119	0	100	0
Pyramimonas parkeae	88	0	120	0	119	0	100	0
Autodesmus obliquus	88	0	120	0	119	0	100	0
Stigeoclonium helveticum	88	0	120	0	119	0	100	0
Trebouxia aggregata	88	0	120	0	119	0	100	0
Volvox carteri	88	0	120	0	119	0	100	0

Table S1. Continued.

	rps18		rps19		tufA		ycf3	
Taxa	aa	missing (%)	aa	missing (%)	aa	missing (%)	aa	missing (%)
Bigelowiella natans	48	0	77	3.8	359	0	161	0
Bryopsis hypnoides	48	0	80	0	359	0	161	0
Chlamydomonas reinhardtii	48	0	80	0	359	0	161	0
Chlorella variabilis	48	0	80	0	359	0	161	0
Chlorokybus atmophyticus	48	0	80	0	359	0	161	0
Coccomyxa subellipsoidea	48	0	80	0	359	0	161	0
Dunaliella salina	48	0	80	0	359	0	161	0
Euglena gracilis	48	0	80	0	359	0	0	100
Floydiella terrestris	48	0	80	0	359	0	161	0
Lepidodinium chlorophorum	48	0	78	2.5	358	0.3	161	0
Leptosira terrestris	48	0	80	0	359	0	161	0
Mesostigma viride	48	0	80	0	359	0	161	0
Micromonas sp. RCC299	48	0	80	0	359	0	161	0
Monomastix sp.	48	0	80	0	359	0	161	0
Nephroselmis olivacea	48	0	80	0	359	0	161	0
Oedogonium cardiacum	48	0	80	0	359	0	161	0
Oltmannsiellopsis viridis	48	0	80	0	359	0	161	0
Oocystis solitaria	48	0	80	0	359	0	161	0
Ostreococcus tauli	48	0	80	0	359	0	161	0
Parachlorella kessleri	48	0	80	0	359	0	161	0
Pedinomonas minor	48	0	80	0	359	0	161	0
Picocystis salinarum	48	0	80	0	359	0	41	74.5
Prasinococcus sp. CCMP1194	48	0	80	0	359	0	161	0
Prasinoderma coloniale	48	0	80	0	359	0	161	0
Prasinophyceae sp. CCMP120[illegible]	48	0	80	0	359	0	161	0
Pseudendoclonium akinetum	48	0	80	0	359	0	161	0
Pycnococcus provasoli	48	0	80	0	359	0	161	0
Pyramimonas parkeae	48	0	80	0	359	0	161	0
Autodesmus obliquus	48	0	80	0	359	0	161	0
Stigeoclonium helveticum	48	0	80	0	359	0	161	0
Trebouxia aggregata	48	0	80	0	359	0	161	0
Volvox carteri	48	0	80	0	359	0	161	0

Table S1. Continued.

Taxa	Total aa	missing (%)
Bigelowiella natans	8585	3.7
Bryopsis hypnoides	8915	0.0
Chlamydomonas reinhardtii	8889	0.3
Chlorella variabilis	8820	1.1
Chlorokybus atmophyticus	8908	0.1
Coccomyxa subellipsoidea	8887	0.3
Dunaliella salina	8889	0.3
Euglena gracilis	8049	9.7
Floydiella terrestris	8613	3.4
Lepidodinium chlorophorum	8835	0.9
Leptosira terrestris	8918	0.0
Mesostigma viride	8905	0.1
Micromonas sp. RCC299	8806	1.3
Monomastix sp.	8712	2.3
Nephroselmis olivacea	8917	0.0
Oedogonium cardiacum	8613	3.4
Oltmannsiellopsis viridis	8917	0.0
Oocystis solitaria	8918	0.0
Ostreococcus tauli	8628	3.3
Parachlorella kessleri	8918	0.0
Pedinomonas minor	8918	0.0
Picocystis salinarum	8758	1.8
Prasinococcus sp. CCMP1194	8844	0.8
Prasinoderma coloniale	8853	0.7
Prasinophyceae sp. CCMP120[illegible]	8869	0.5
Pseudendoclonium akinetum	8918	0.0
Pycnococcus provasoli	8760	1.8
Pyramimonas parkeae	8696	2.5
Autodesmus obliquus	8807	1.2
Stigeoclonium helveticum	8611	3.4
Trebouxia aggregata	8841	0.9
Volvox carteri	8871	0.5

Table S2. Codon frequency[1] and tRNA genes encoded in *Lepidodinium chlorophorum* plastid genome

Codons	AA	Freq (‰)	tRNA	Codons	AA	Freq (‰)	tRNA	Codons	AA	Freq (‰)	tRNA	Codons	AA	Freq (‰)	tRNA
UUU	F	58.59	GAA	UCU	S	25.93	GGA	UAU	Y	25.8	GUA	UGU	C	8.73	GCA
UUC		11.10		UCC		6.67		UAC		9.31		UGC		3.40	
UUA	L	2.57	CAA	UCA		1.03	CGA	UAA	*	2.82	—[3]	UGA	*	0.39	—[3]
UUG		36.07		UCG		15.27		UAG	*	0.71		UGG	W	16.94	CCA
CUU		26.95	UAG	CCU	P	17.01	UGG	CAU	H	15.92	GUG	CGU	R	17.58	ACG
CUC		6.48		CCC		2.89		CAC		6.42		CGC		6.48	
CUA		19.38		CCA		14.57		CAA	Q	32.41	UUG	CGA		18.87	
CUG		7.19		CCG		4.43		CAG		6.16		CGG		3.02	
AUU	I	54.74	GAU	ACU	T	18.74	UGU	AAU	N	35.81	GUU	AGU	S	12.26	GCU
AUC		13.09		ACC		4.88		AAC		15.02		AGC		5.58	
AUA	M	28.62	CAU[2]	ACA		26.50		AAA	K	52.56	UUU	AGA	R	15.66	UCU
AUG		14.57		ACG		6.55		AAG		9.88	CUU	AGG		3.40	
GUU	V	27.66	UAC	GCU	A	18.93	UGC	GAU	D	24.84	GUC	GGU	G	23.49	UCC
GUC		5.84		GCC		4.24		GAC		7.64		GGC		4.68	
GUA		20.86		GCA		22.53		GAA	E	35.81	UUC	GGA		28.94	
GUG		7.44		GCG		6.23		GAG		7.12		GGG		4.81	

[1]This analysis included functionally unassigned open reading frames (URFs) and excluded the supposed pseudogene of *rpoC1*.
[2]Both methionyl-tRNA genes for initiation and elongation were found.
[3]No tRNA for termination codons.
