# Supplementary material for: Plastid Genome-Based Phylogeny Pinpointed the Origin of the Green-Colored Plastid in the Dinoflagellate Lepidodinium chlorophorum
Source: Genome Biol Evol. 2015 Apr 2;7(4):1133–40. doi: 10.1093/gbe/evv060 (PMC4419806; doi:10.1093/gbe/evv060)
Supplement: Supplementary Data [file supp_evv060_New_Microsoft_Office_Word_Document.docx]

**Fig. S1**

**Fused ORFs.** A. *rpl19*-*atpA*. B. *psbK*-*rpl20*. *rpl19* and *psbK* regions are highlighted in red, while *atpA* and *rpl20* regions are highlighted in blue. Termination codons were indicated with asterisks.

**Fig. S2**

**Physically overlapping gene pairs.** A. *rpl23-rpl2*, B. *rpl14-rpl5*, C. *petL-petG*, D. *atpI-rpoC1*, E. *rps11-rpoA*, F. *psaC-trnK*(*cuu*), G. *psbD-psbC*, H. *psbB-psbT*, I. *psbH-urf129*. Nucleotide sequences shared by two distinct genes are highlighted in black. The deduced amino acid sequences are shown below the nucleotide sequences. The nucleotide sequences for *trnK*(*cuu*) are italicized in F. The coding region of *psbC* likely starts with a GUG codon (see F). Termination codons are indicated by asterisks.

**Fig. S3**

**Introns.** We predicted the insertion sites, 5′ and 3′ boundary sequences, and secondary structures at the 3′ termini of *petD* intron (A), *psbA* intron (B), and *psbB* intron (C). From all the three introns, group II intron-specific stem-loop structures were predicted (i.e. domains V and VI). Especially, domain V is highly conserved amongst group II introns, as this region forms a part of the catalytic sites for splicing reaction (Lambowitz and Zimmerly 2004). Asterisks indicate the putative branch-point adenosines in domain VI that enable the excised introns to form the characteristic lariat-shape structure (Lambowitz and Zimmerly 2004). We supply the amino acid sequences deduced from the *Lepidodinium chlorophorum* nucleotide sequences flanking to the introns, as well as the corresponding amino acid sequences of the green algal homologues.

**Fig. S4**

**Nucleotide alignment of *ycf3* and ψ*ycf3* regions.** Identical nucleotides are indicated by asterisks. Initiation and termination codons are shaded in grey and black, respectively.

**Fig. S5**

**Nucleotide sequence and deduced amino acid sequence of *rpoC1* in the *Lepidodinium chlorophorum* plastid genome.** The amino acid sequence of the *Pedinomonas minor* homologue is aligned with that of the *L. chlorophorum* homologue. In-frame termination codons in *L. chlorophorum rpoC1* are shaded in red. Identical and similar amino acids between the two homologues are shaded in black and grey, respectively.

**Fig. S6**

**Maximum likelihood tree inferred from a 32-taxon 52-protein alignment.** The alignment comprises 52 plastid proteins (8,917 amino acid positions in total) shared among 32 plastid genomes of two streptophytes, 10 prasinophytes, 17 core chlorophytes, *Euglena gracilis*, *Bigelowiella natans*, and *Lepidodinium chlorophorum*. ML bootstrap supports equal to or greater than 80% are shown on each node.
